# Supplementary material for: Structural basis for the recognition of LDL-receptor family members by VSV glycoprotein
Source: Nat Commun. 2018 Mar 12;9:1029. doi: 10.1038/s41467-018-03432-4 (PMC5847621; doi:10.1038/s41467-018-03432-4)
Supplement: Supplementary file 1 — Supplementary Information [file 41467_2018_3432_MOESM1_ESM.pdf]

Supplementary Information

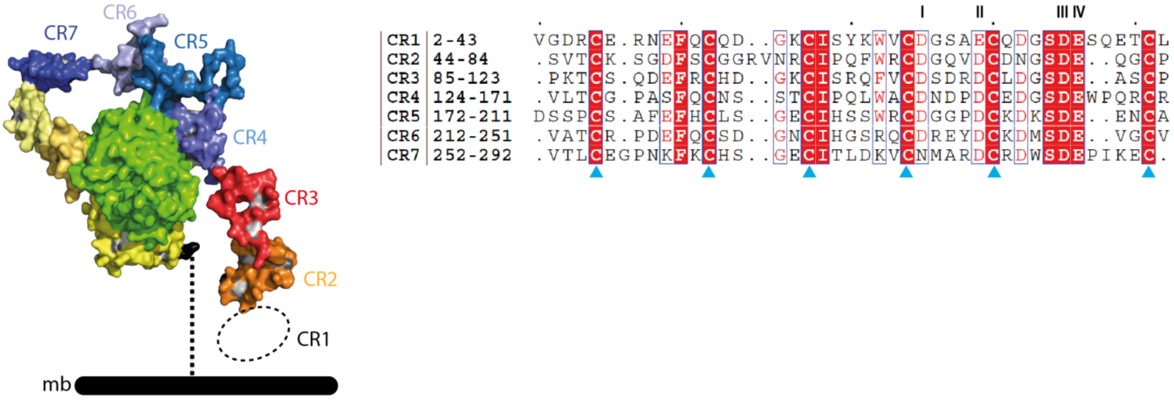

Supplementary Figure 1 (related to the introduction):

Crystal structure of the LDL-R ectodomain (from CR2 to the EGF domain) in its closed conformation at acidic pH [1] and sequence alignment of the CR repeats of the human LDL-R. Acidic residues involved in the Ca^{2+} cage are labelled with I II III and IV, conserved cysteine residues engaged in disulfide bonds are marked by a blue triangle. Conserved residues are in red boxes and similar residues are shown by red letters boxed in blue.

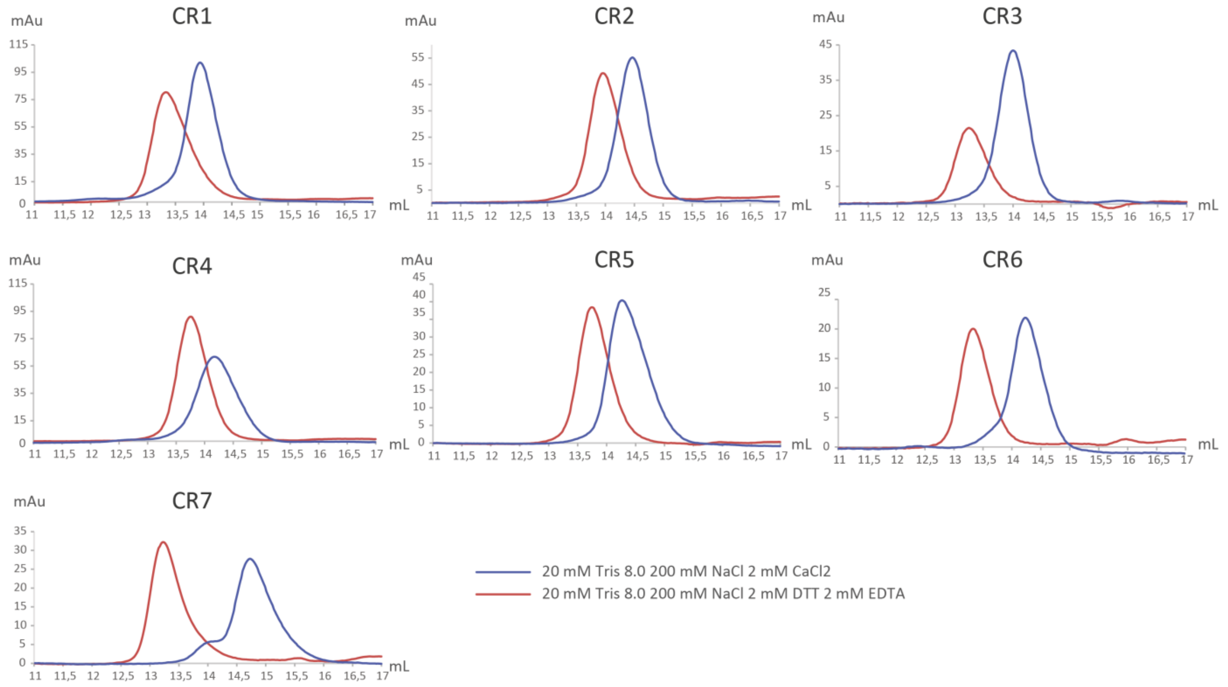

Supplementary Figure 2 (Related to figure 1 and 2):

Gel filtration elution profile of purified CR domains on a S75 Superdex HR10/30. Elution profiles of refolded CR domains are in blue and of CR domains after denaturation by 2 mM EDTA and 2 mM DTT are in red. Refolded CR domains eluted at 14.25 +/- 0.25 ml and denatured CR domains eluted at 13.5 +/-0.25 ml.

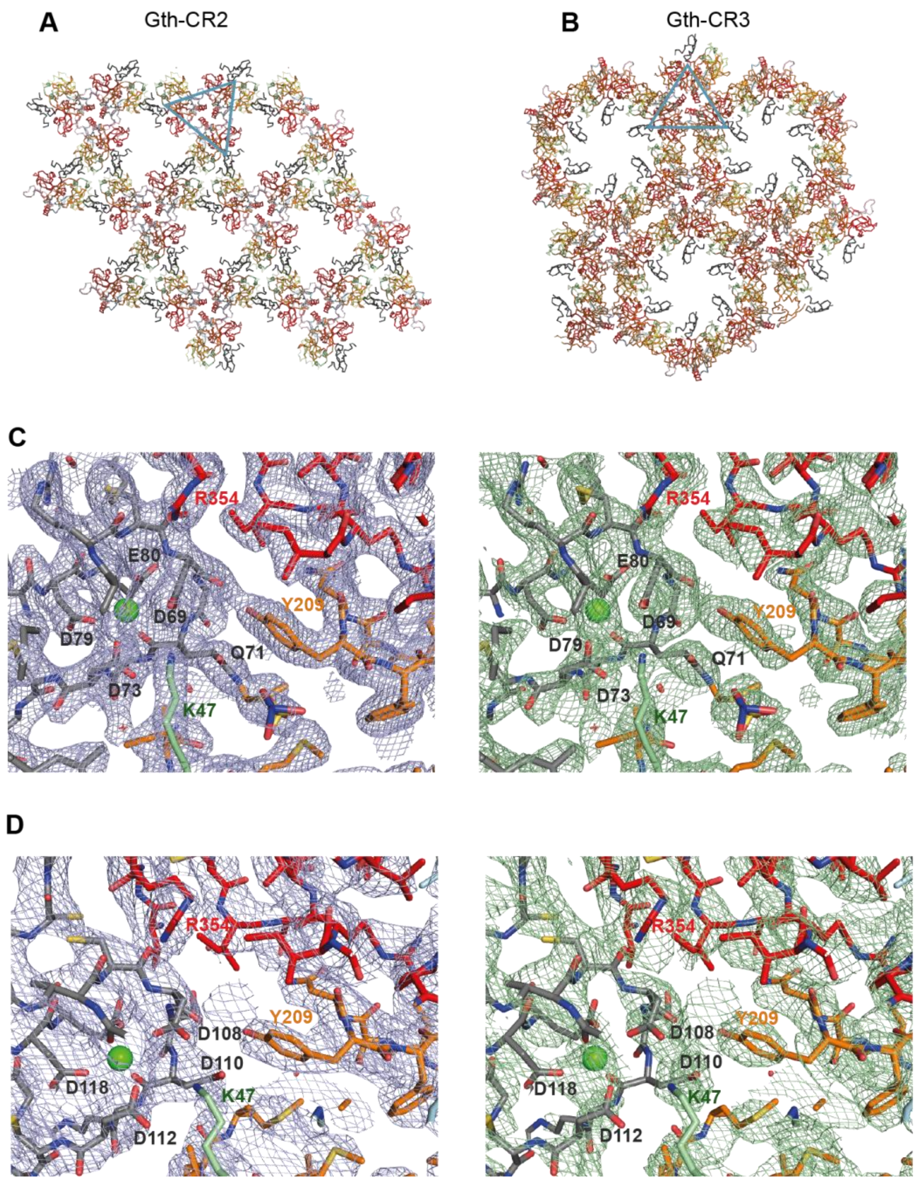

Supplementary Figure 3 (Related to figure 3 and 4):

A Crystal lattice of G_{th}-CR2 crystals belonging to the space group *H*32.

B Crystal lattice of G_{th}-CR3 crystals belonging to the space group *P*622.

G is colored by domains and CR are in black. In **A** and **B,** the blue triangle indicates a G a pre-fusion trimer.

C-D Interaction area of both G_{th}-CR2 (**C**) and G_{th}-CR3 (**D**) models represented in sticks and accompanying electron density maps. Key residues discussed in the text are indicated.CR

domains are in grey and G is depicted by domains. The final refined $2F_O$-F_C map (in blue) and omit map (in green) are both contoured at 1σ. Omit map to validate the agreement between the atomic model and X-ray data was calculated using SFCHECK[2].

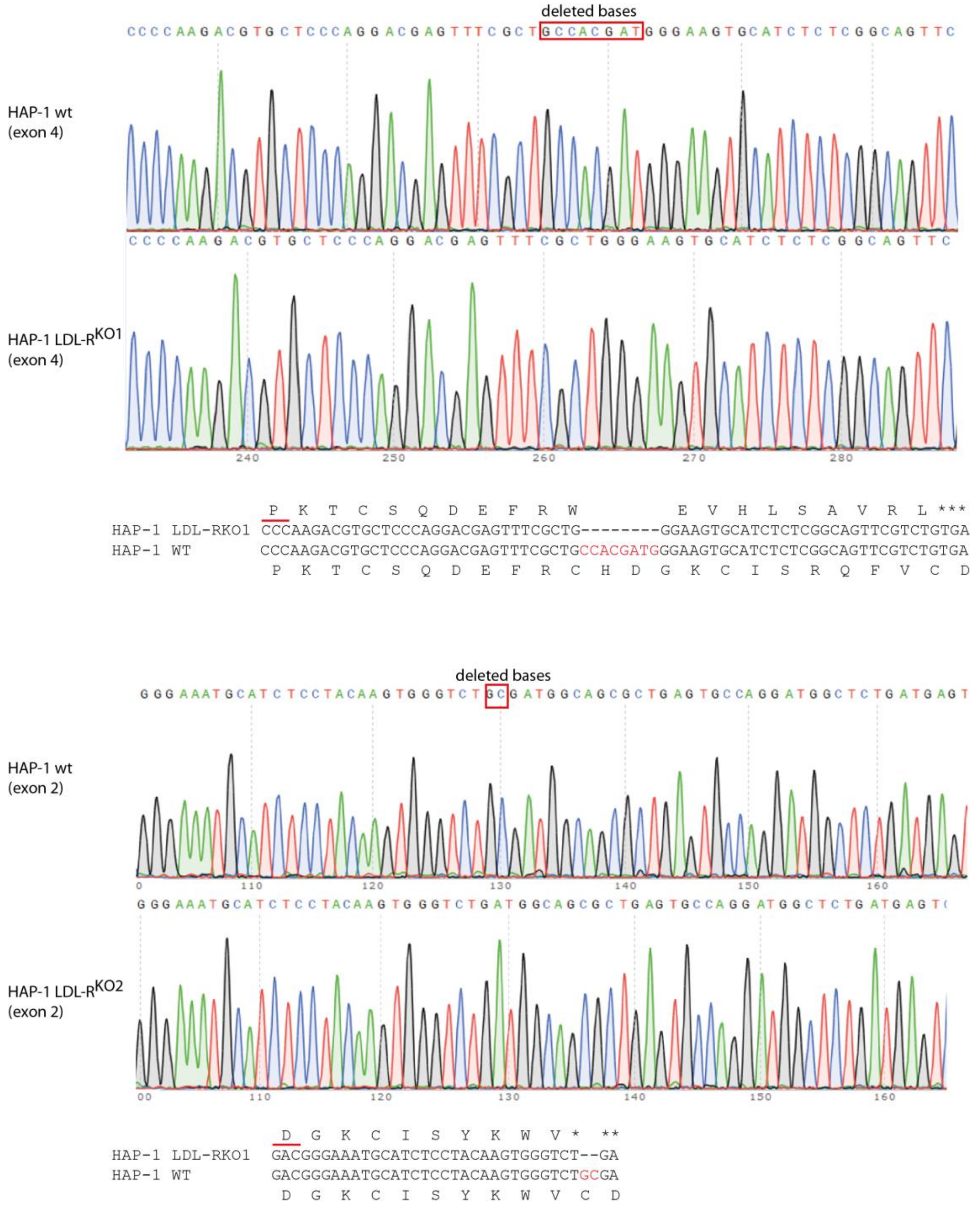

Supplementary Figure 4:

Nucleotide sequence of the regions flanking the deletion in the LDL-R gene in HAP-1 LDL-R^{KO1} and HAP-1 LDL-R^{KO2} cell lines. The protein sequence is indicated above the nucleotide sequence. The deleted nucleotides are in red. The first stop codon following the deletion is indicated by three stars.

Supplementary Table 1: Data collection and refinement statistics

	Crystal G_{th}CR2 (pdb code 5OYL)	Crystal G_{th}CR3 (pdb code 5OY9)
**Data collection**		
Space group	H32	P622
Cell dimensions		
a, b, c (Å)	90.04 90.04 515.78	90.00 90.00 120.00
α, β, γ (°)	90.00 90.00 120.00	122.35 122.35 197.81
Resolution (Å)[a]	49.68-2.24 (2.30-2.24)	46.70-3.60 (3.73-3.6)
R_{merge}	0.184 (3.861)	0.278 (5.096)
R_{meas}	0.203 (4.242)	0.298 (5.469)
R_{pim}	0.084 (1.741)	0.106 (1.957)
$I/\sigma(I)$	8.7 (1.61)	9.2 (1.32)
$CC_{1/2}$	0.998 (0.494)	0.998 (0.222)
Completeness (%)	99.9 (98.3)	99.8 (98.1)
Redundancy	11.1 (10.9)	13.8 (13.7)
**Refinement**		
Resolution (Å)	29.98 - 2.25	44.85 - 3.6
No. Reflections	29837	10727
R_{work}	0.190	0.236
R_{free}	0.219	0.268
No. atoms		
Macromolecules	3567	3525
Ligand/ion	96	56
Water	149	
B-factors		
Macromolecules	53.32	156.42
Ligand/ion[b]	82.93	179.35
Solvent	45.96	116.86
R.m.s. deviations		
Bond lengths (Å)	0.007	0.006
Bond angles (°)	0.98	0.83

[a] The values in parentheses are for the highest-resolution shell.

[b]Ligand/ion includes a Ca atom in the acidic cluster of both CR domains.

Supplementary Table 2: primers used for RT-PCR experiments

mRNA	Primers	Expected size
vLDLR	ACATGAAATCAGCTGTGGCG<br>AGTCTGCATCATCGTCGCAT	308
LRP1	GAACCCATCCTACGTGCCTC<br>CAGTCATTGTCATTGTCGCATCT	474
LRP2	GTGCAGACCTAAAGGAGCGT<br>ACAACAGCGCAGCCAATTTC	300
LRP3	AACCAGAAAAGCTGTCCCGA<br>GGTCTCGAAGGCCCTGTATT	291
LRP4	GTGCAGTGAGTGCTCTTGGA<br>CGCATGTCACACTGCTCATC	337
LRP5	GTCGTCGGTGACAGAGTTACA<br>TCTCGAGGGAGATCCTGTGG	384
LRP6	ATTATTGTCCCCCGATGGGC<br>TAACCACTGCCTGCCGATTT	383
LRP8	GACGACGACTGTGGTGACG<br>TGGTTGCAGTGCTTGATTGC	395
SORL1	CAACTGCGAAAACCCCACAG<br>TACACGTCTTCGGTTGGTGG	383

Supplementary Reference

1. Rudenko, G. et al. Structure of the LDL receptor extracellular domain at endosomal pH. *Science* **298**, 2353-8 (2002).

2. Vaguine, A. A., Richelle, J. & Wodak, S. J. SFCHECK: a unified set of procedures for evaluating the quality of macromolecular structure-factor data and their agreement with the atomic model. *Acta Crystallogr. D Biol. Crystallogr*. **55,** 191–205 (1999).
